# Dynamics of Lateral Habenula–Ventral Tegmental Area Microcircuit on Pain-Related Cognitive Dysfunctions

**DOI:** 10.3390/neurolint15040082

**Published:** 2023-10-27

**Authors:** Ana Raquel Pereira, Mobina Alemi, Mariana Cerqueira-Nunes, Clara Monteiro, Vasco Galhardo, Helder Cardoso-Cruz

**Affiliations:** 1 Instituto de Investigação e Inovação em Saúde—Pain Neurobiology Group, Universidade do Porto, Rua Alfredo Allen 208, 4200-135 Porto, Portugal; aramaralpereira@gmail.com (A.R.P.); mobinaalemi@yahoo.com (M.A.); mariana.nunes@i3s.up.pt (M.C.-N.); cmonteir@med.up.pt (C.M.); galhardo@med.up.pt (V.G.); 2 Instituto de Biologia Molecular e Celular, Universidade do Porto, Rua Alfredo Allen 208, 4200-135 Porto, Portugal; 3 Departamento de Biomedicina—Unidade de Biologia Experimental, Faculdade de Medicina, Universidade do Porto, Rua Doutor Plácido da Costa, 4200-450 Porto, Portugal; 4 Programa Doutoral em Neurociências, Faculdade de Medicina, Universidade do Porto, Rua Doutor Plácido da Costa, 4200-450 Porto, Portugal

**Keywords:** lateral habenula, ventral tegmental area, chronic pain, cognitive dysfunctions, mood disorders

## Abstract

Chronic pain is a health problem that affects the ability to work and perform other activities, and it generally worsens over time. Understanding the complex pain interaction with brain circuits could help predict which patients are at risk of developing central dysfunctions. Increasing evidence from preclinical and clinical studies suggests that aberrant activity of the lateral habenula (LHb) is associated with depressive symptoms characterized by excessive negative focus, leading to high-level cognitive dysfunctions. The primary output region of the LHb is the ventral tegmental area (VTA), through a bidirectional connection. Recently, there has been growing interest in the complex interactions between the LHb and VTA, particularly regarding their crucial roles in behavior regulation and their potential involvement in the pathological impact of chronic pain on cognitive functions. In this review, we briefly discuss the structural and functional roles of the LHb–VTA microcircuit and their impact on cognition and mood disorders in order to support future studies addressing brain plasticity during chronic pain conditions.

## 1. Introduction

Chronic pain and depressive conditions are frequently encountered in clinical practice, making the successful treatment of pain in patients more challenging. Specifically, depressive symptoms can prolong the duration and increase the intensity of pain [1]. This often establishes a cyclic relationship between pain and depressive symptoms, which significantly impacts executive and cognitive functions [2,3,4]. One critical contributor to the pain experience is the lateral habenula (LHb), which is known to be activated by aversive states, including chronic stress-related anxiety [5] and pain [2]. The LHb plays a role in the pathogenesis of pain by participating in pain transmission [6], modulating pain intensity [7], and contributing to the emotional component of pain [8]. Moreover, the LHb is closely associated with the processing of reward information and cognitive flexibility [9,10]. Several LHb-dependent responses to adverse events are mediated through their effects on neurons in the ventral tegmental area (VTA). The VTA is primarily composed of dopaminergic (DAergic) neurons [11,12] and is involved in important processes that support motivational and cognitive components [13,14]. Additionally, this area is associated with the encoding of aversive stimuli, including painful stimuli [15,16]. For instance, it has been shown that VTA lesions increase pain-related behavioral responses [17] and pain sensitivity [18]. At the structural level, the LHb and VTA brain areas share bidirectional synaptic interactions. The LHb exerts an inhibitory effect on the VTA through its glutamatergic neurons acting on local VTA GABAergic neurons; thus, it functions as an inhibitory tone for the VTA dopamine (DA) pathways [19,20]. In fact, it has been shown that in chronic pain conditions, there is a dysregulation in DA transmission [21]. One hypothesis to explain this dysregulation postulates that deficient DA signaling related to pain can result from local hyperactivity of the LHb [19,22]. As VTA DA neurons project to several areas through the mesocortical and mesolimbic pathways and play important roles in cognition, motivation, and reward [2,23,24], this impairment can be amplified. Consequently, the disruption or manipulation of LHb–VTA activity can have a major impact on pain-related cognitive functioning. This review aims to gather information about this microcircuit. Firstly, we provide an update on the basic organization of the LHb and VTA, along with a discussion of their roles in pain and cognitive information processing. Secondly, we describe the various functions that have been attributed to the structural connectivity between the LHb and VTA. In this review, we emphasize the dissection of studies highlighting the specific functions of the upstream input and downstream output pathways of the LHb and VTA, as well as the dependent relationship between them. Thirdly, we examine the relevance of the LHb–VTA microcircuit in major cognitive components. We focus on the functional and behavioral mechanisms leading to aberrant overactivation of this microcircuit in preclinical and clinical studies. This discussion leads to an exploration of potential strategies that may specifically target the neural properties of this microcircuit in pain-related impairments.

## 2. Lateral Habenula

**Figure 1:**
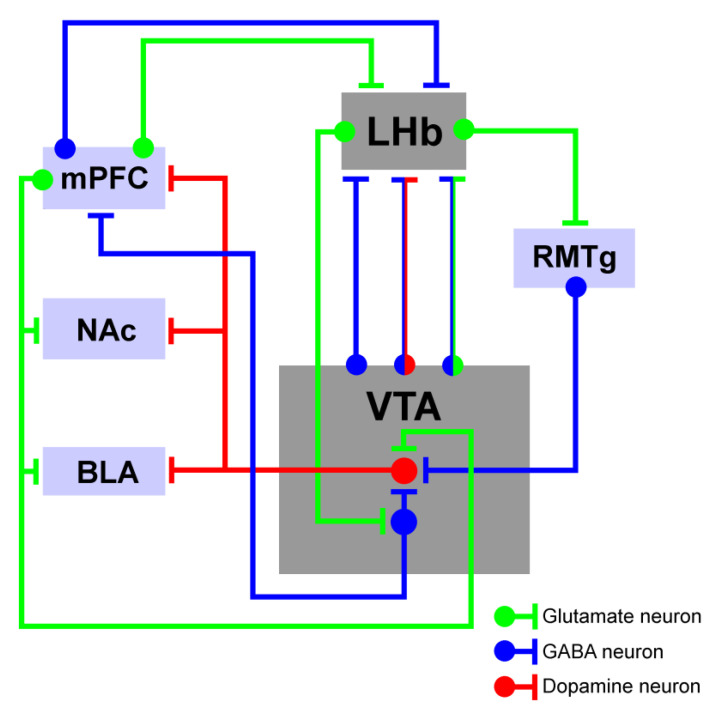
Intra-hemispheric representation of the lateral habenula and ventral tegmental area microcircuit inputs and outputs with their respective neurotransmitter systems. BLA, basolateral amygdala; LHb, lateral habenula; mPFC, medial prefrontal cortex; NAc, nucleus accumbens; RMTg, rostromedial tegmental area; and VTA, ventral tegmental area.

Due to its anatomical architecture and interactions between midbrain and forebrain areas (Figure 1), the LHb has been associated with a wide range of complex cognitive functions and several brain disorders and dysfunctions. In the literature, the LHb has been reported as being involved in pain processing [25,26,27], stress [5,28], depression, and pain-related depression [2,29,30]. Regarding the mood component, LHb activity plays an important role in motivation [31], emotion [32], impulsivity, and aggressive behaviors [33]. It also contributes to higher cognitive processes, such as spatial memory [34], working memory [35], and reward-related decision making [36,37]. Additionally, the LHb is considered to be a hub for anti-reward responses due to its activation in response to expectancy, aversive stimuli, or even the omission of expected rewards [9,36,38,39]. LHb neurons can encode reward prediction errors by adjusting their activity in response to expected and actual rewards, enabling more efficient adaptation of behavior and guiding future reward-related actions [9]. The LHb has also been shown to contribute to circadian timekeeping [40], fear behavior [41], regulation of feeding behavior [42], brain state transitions during coping behaviors [43], and the regulation of anxiety- and panic-related defensive responses [44].

### 2.1. The Role of the LHb in Pain Processing

LHb involvement in pain information processing is facilitated through direct afferent inputs originating from the dorsal horn lamina I [6], trigeminal nucleus [6], and hypothalamus [45]. It plays a pain modulatory role through indirect pathways involving the midbrain central gray and serotoninergic raphe nuclei, which typically convey painful or analgesic information [22,46]. Early studies provided the first evidence of this by showing that direct electric stimulation of the habenular complex can induce analgesia [27,47]. In rodents, neuroelectrophysiological recordings have demonstrated that LHb neurons respond to noxious stimuli, but not to non-noxious stimuli [48]. Moreover, it has also been reported that a significant number of LHb neurons change their activity during pain conditions [1,49]. These observations are supported by clinical imaging studies, which have shown LHb activation during acute pain [26], and chronic pain conditions [50]. Furthermore, the activation of local LHb neurons through L-glutamate injection has demonstrated an important decrease in pain thresholds [51]. In addition, increased c-fos activity has been reported in the LHb during acute pain experiences [52] and the recall of past painful episodes [8]. More recently, a higher LHb c-fos activity was observed in rats subjected to a model of chronic unpredictable mild stress; this was accompanied by depressive behaviors [1]. Li and colleagues [1] showed that when this stress model was combined with inflammatory pain (formalin injection), the LHb activity was even higher, suggesting a synergistic effect between depression and pain. Notably, they also found that selective LHb lesions reversed depression symptoms and hyperalgesia in a rodent model of chronic pain [1,2]. Furthermore, increased LHb activity was reported during the withdrawal phase of alcohol consumption and was usually associated with a hyperalgesic state [7]. The chemogenetic inhibition of LHb excitatory neurons reduced this hyperalgesia, while LHb chemogenetic activation induced a hyperalgesic state in naive rats in response to thermal stimulation [7]. The function of the LHb is intricately connected to its densely interwoven areas [26,53]. In addition to the VTA, the LHb also receives projections from the locus coeruleus, which is responsible for releasing norepinephrine (NE) within the LHb, contributing to the induction of anxiogenic behaviors. For instance, the intra-LHb injection of dexmedetomidine, an α_2_ adrenoreceptor agonist, produces sedative and anxiolytic effects [54]. Given that pain is typically associated with arousal and anxiety, the role of NE in the LHb may also be significant in the processing of pain-related emotions.

In line with the preclinical data, human imaging studies have shown that the habenula complex is either activated or exhibits abnormal disturbances of its function in chronic pain patients [55] and depressive patients [56,57]. Notably, imaging studies in humans have demonstrated habenula activation in response to noxious heat [26]. Reduced habenula volume has also been reported using postmortem histological analyses of human brain tissue [58] and structural MRI recordings in patients with major depressive disorder and bipolar disorder [59,60]. Moreover, a PET study has shown enhanced coupling between the habenula and raphe nuclei in patients experiencing transient depressive relapse upon tryptophan depletion [61]. An increased theta and alpha oscillatory synchrony in the fronto-habenular network has also been associated with negative emotional valence in human patients [62]. These findings suggest that high-frequency, deep-brain stimulation of the habenula may offer benefits in the treatment of resistant major depressive disorder by disrupting the information flow from the prefrontal cortex to the habenula complex. More recently, it has also been demonstrated that chronic low back pain is associated with abnormal resting-state functional connectivity and effective connectivity of the habenula [63]. However, the underlying mechanisms of these alterations remain largely unknown.

### 2.2. The Role of the LHb in Mood and Cognition

The LHb has also been investigated with regard to its role in important emotional and cognitive functions. From a structural point of view, the LHb integrates data from the limbic system and basal ganglia, relaying this information to mesolimbic areas that contribute to the selection of appropriate behaviors and to a flexibility of choices [37,64]. Extracellular neuroelectrophysiological recordings conducted simultaneously in the LHb and the hippocampal formation of anesthetized and freely moving rats, have shown that LHb neurons are phase-locked with hippocampal theta oscillations during the performance of spatial recognition tasks. Silencing LHb activity during these tasks can lead to a reduction in behavioral accuracy [4]. In humans, it has been demonstrated that habenula high-gamma activity increases during the receipt of loss and decreases during the receipt of reward [65]. The lesion or temporary inactivation of the LHb has been associated with learning deficits during the forced-swim test [66,67,68], impaired spatial reference memory in the Morris water maze test [69], and attentional deficits characterized by marked premature response in the five-choice serial reaction time task [70]. Additionally, the LHb complex has been implicated in the long-term storage of aversive memories [71]. On the other hand, it is important to point out that the outcomes of LHb manipulation may vary depending on the specific experimental model applied. For example, rats with lesions to the medial forebrain bundle, which typically exhibit working memory deficits, showed increased dopamine (DA) levels in the medial prefrontal cortex (mPFC), hippocampus, and amygdala after LHb lesions [72]. Surprisingly, this increase in DA levels improved their working memory performance in a T-maze rewarded alternation task [72]. Similarly, the pharmacological inactivation of the LHb has been associated with abnormal responses during the initial stages of memory formation and during the retrieval phase in the Morris water maze test [34]. However, the consolidation of spatial memory does not appear to be affected [34]. In fact, the LHb complex appears to play a plausible role in working memory as it receives afferents from the mPFC [35,73], a brain region also affected by chronic pain conditions [10,74,75,76]. Furthermore, disruptions in local LHb activity have been associated with deficient decision-making performance in a repeated probabilistic reversal task [77]. This impairment appears to be directly dependent on DA neuron activity [77]. Notably, after LHb inactivation, rats were observed to select their choices without considering the reward magnitude or the cost of obtaining it. This highlights the important role of the LHb in the behavioral flexibility necessary for successful goal-directed tasks (for a review, see Baker and Mizumori [10]). Regarding the ability to adapt and change behavioral responses to unpredictable events, both DA and LHb have been implicated in this process. For instance, increased DA release in the mPFC has been observed during the learning of spatial reversal learning tasks [78]. However, using the same behavioral task and after pharmacological LHb inactivation, Baker and colleagues reported a significant impairment of the behavioral performance [79]. The LHb is involved in reward and aversion and is reciprocally connected to DAergic areas, including the VTA [80]. It serves as the primary inhibitor of VTA activity [19,81]. The modulation of DA receptors has been shown to offer a specific strategy for altering pain sensation by changing neuronal excitability and synaptic transmission. Two comprehensive reviews on the topic can be found in [82,83]. Collectively, these studies suggest the potential involvement of the LHb–VTA microcircuit in these processes.

## 3. Ventral Tegmental Area

Given its cell-type architectural composition, the VTA is a heterogeneous brain area. Most of the VTA neurons are DAergic and GABAergic. Glutamatergic neurons are also present, but in lower numbers [84]. The exact distribution and percentages of these three types of neurons is not entirely clear because some of the DAergic neurons can co-express and release other neurotransmitters such as GABA and glutamate [85,86], depending on their projection targets [87]. Furthermore, this complexity is heightened by the fact that VTA DA neurons exhibit heterogeneity not only in their anatomical and molecular characteristics but also in their electrophysiological activity patterns [88,89,90,91]. The neuroelectrophysiological properties of VTA neurons, which are commonly used to distinguish DA from non-DA neurons, vary depending on their projection targets (Figure 1). For instance, while DA neurons typically display slow firing properties, DA neurons that specifically target mPFC and nucleus accumbens (NAc, both core and medial shell regions) show atypical fast firing rates [89]. At the behavioral level, VTA activity contributes to several behaviors and complex cognitive functions, including adaptive behaviors (flexibility and reinforcement learning), working memory, motivation, aversion, and the encoding of value and salience [92,93,94]. Recent research has demonstrated that VTA DA neurons are not only involved in assessing the value and salience of stimuli but also in their identification [95]. Serving as a central hub for reward information processing, the VTA plays a key role in reward-based learning and goal-directed behaviors [94]. Additionally, the VTA is also known to be involved in certain pathological states, such as depression [96], addiction [97], and schizophrenia [98]. The VTA influences these processes by establishing connections and communication between multiple brain areas, primarily through the release of DA in limbic and cortical areas, and thereby modulates the activity of neurons in those regions [99,100].

### 3.1. The Role of the VTA in Pain Processing

Several studies have explored the contribution of the VTA in nociception modulation. The initial studies revealed that in response to aversive or painful stimuli, some VTA DA neurons increase their firing activity, while others decrease it [101,102]. In a study by Ungless and colleagues [103], it was found that aversive stimuli uniformly inhibit VTA DA neurons. However, they also reported an excitatory effect in neurons lacking tyrosine hydroxylase (TH) expression [103]. Similarly, Brischoux and colleagues [92] observed that following a painful stimulus, most of the VTA TH-positive DA neurons located in the ventral part of the VTA were inhibited, while some were unresponsive and others were strongly excited [92]. Another important point is the direct and/or indirect unbalanced interaction between the VTA DAergic tone and other brain areas. For example, acute pain can activate DAergic signaling in PFC areas [104], whereas chronic pain conditions may reduce DAergic signaling, probably due to disruptions in local VTA networks [105]. Following the induction of peripheral neuropathy, a significant decrease in VTA c-fos activity was reported 4 days later, supporting the hypothesis regarding a hypo-DA activity pattern during chronic pain [106]. However, in another study, the authors observed an increase in VTA DA bursting activity and a decrease in the evoked inhibitory input from the rostromedial tegmental area (RMTg) 14 days after nerve lesion [107]. Although the spontaneous activity of the RMTg remained unaffected, the study reported an increase in extracellular DA levels and a decrease in the expression of TH and DA D2 receptor (D2r) protein in the NAc. This increase in DA activity appears to contradict the findings of other studies, which reported a hypo-DA tone in chronic pain conditions (for a review, see [82,108,109,110]). One possible explanation for this discrepancy could be a compensatory mechanism, whereby DA cells attempt to cope with the negative experiences [111]. Additionally, a recent study showed that neuropathic pain can lead to differential plasticity in specific DA neurons located in lateral and medial regions of the VTA [112]. They observed a significant decrease in DA activity in the lateral VTA, but not in the medial VTA [112]. All these studies emphasize the importance of subpopulation specificity during attempts to modulate VTA DA neurons, particularly in painful conditions. Notably, VTA DAergic signaling seems to develop a vital influence in the specific synergy between the VTA and other brain regions during pain states. In a rodent model of neuropathic pain, it has been reported that reduced intrinsic excitability of VTA DA neurons contributes to decreased NAc local firing activity [113]. This disruption can be reverted by selective optogenetic activation of NAc-projecting VTA DA neurons, reducing the allodynia and hyperalgesia caused by sciatic nerve lesion [113]. Adding to this, it has also been reported that VTA stimulation can reduce thermal and mechanical responses and spinal dorsal horn excitability induced by inflammatory pain [114]. Furthermore, DA neurons also play a critical role in the emotional component of pain. As mentioned before, pain relief in the context of ongoing pain can lead to an increase in VTA DA neuron activity [90,92]. This results in a conditioned place preference as to where pain relief is applied, reflecting the rewarding effect of VTA DA activation in the affective pain component [115]. Furthermore, opioids also play a role in VTA signaling in pain, since opioids hyperpolarize GABA interneurons in the VTA leading to an increase in VTA DA neuron activity [116]. This accounts for the analgesic effect observed following opioid administration and the release of high-levels of DA from VTA terminals in the NAc [117]. However, it is important to note that opioid administration does not appear to contribute to the direct rewarding dimension of pain relief, as the conditioned place preference for pain relief does not depend on the direct VTA opioid effect [115].

### 3.2. The Role of the VTA in Mood and Cognition

Dopamine plays a pivotal role in different cognitive functions, including working memory [118,119], adaptive behaviors [120,121], incentive learning [122], value-based learning [123], decision making [124,125], motivation [126], valuation [127], cognitive control [128], and action initiation [129,130]. Consequently, the VTA with its majority of DA neurons is either directly or indirectly involved in these cognitive functions [131]. Many of these functions rely on the role of DA neurons in encoding reward prediction errors [132] and valence, which affect decision-making processes [133]. VTA DA neurons are believed to contribute significantly to cognition through their connections to cortical areas, particularly through their indirect control of mPFC neurons [134,135]. As several cognitive functions are dependent on mPFC functioning [136], the bidirectional interplay between the VTA and mPFC allows for adjustments in VTA DA signaling to the mPFC. This coordination of neuronal activity is essential for meeting cognitive demands [137,138]. In this regard, the overexpression of DA D2r in the striatum has been associated with a disruption of neuronal activity coordination between the VTA and mPFC, affecting the learning rate in spatial working memory tasks [99]. Another important component is intra-VTA oscillatory activity and its synchronization with the hippocampal formation and the mPFC activity, which has also been referred to as being key to the success of working memory-dependent processes [139]. It is important to note that VTA DA neurons form a functional loop with the hippocampus [140]. When the hippocampus detects new information, this loop is activated and the resulting novelty signal is conveyed to the VTA DA neurons, leading to their firing. This, in turn, causes a release of DA within the hippocampus, enhancing long-term potentiation (LTP) and learning [140]. On the other hand, VTA inactivation has also been shown to suppress LTP in the hippocampal CA1 field [141]. Finally, it is important to note that it is not only VTA DA neurons that are involved in cognitive processes; the local VTA glutamatergic neurons also play an important role. For example, the brief photo-stimulation of VTA VGlut2 positive neurons has been shown to induce positive reinforcement in instrumental behavioral assays, while their continuous stimulation demonstrated an opposite result with the inducing of avoidance responses [87].

## 4. LHb-to-VTA Pathway Structural Connectivity

The VTA is one of the main efferents of the LHb (Figure 1). These projections are mainly glutamatergic [142,143] and exert an inhibitory tone over the VTA GABAergic interneurons that further suppress the local DA neuron activity [19]. In turn, the VTA also sends projections back to the LHb. This occurs mainly through its GABAergic, GABAergic-glutamatergic [87,144], and GABAergic-DAergic neurons [145], which connect the VTA to local LHb glutamatergic neurons. The LHb and VTA are also anatomically connected by indirect pathways. The main indirect pathway from the LHb to the VTA is through the RMTg. In this case, the LHb glutamatergic projections form synapses on the local RMTg GABAergic neurons, and they in turn exert an inhibitory effect over the local VTA DA neurons [146]. From the VTA, there are also some indirect pathways to the LHb going through different brain structures, such as the ventral striatum or the PFC [147,148]. Together, these microcircuits are important for information segregation to other brain regions.

### 4.1. The Influence of LHb-Dependent Activity on the VTA

Several neuroelectrophysiological studies have shown that the inhibitory input from the LHb to the DA neurons is undoubtedly robust [19,20,149]. For example, electrical stimulation of the LHb in anaesthetized rats showed that 97% of the VTA DA neurons developed transient inhibition [19]. However, it is important to note that the RMTg also appears to contribute to this input. It has been shown that when combining local stimulation of the LHb with local RMTg lesions, only 67% of the VTA DA neurons were inhibited, suggesting that the RMTg relays a part in the inhibitory tone sent by the LHb to the VTA [149]. Although most of the VTA DAergic population is inhibited following LHb excitation, there are also some VTA DAergic neurons that increase their activity upon LHb stimulation [38,39]. It has been suggested that direct contact between the LHb glutamatergic axon terminals and local VTA DAergic neurons seems to be responsible for this particular enhanced activity [142,150]. In turn, LHb inhibition or direct stimulation of the VTA has been shown to increase extracellular DA concentration in the mPFC and ventral striatum (NAc) [151].

### 4.2. The Influence of VTA-Dependent Activity on the LHb

The VTA also exerts a modulatory effect on local LHb activity. For example, it has been shown that the optogenetic selective activation of VTA glutamatergic neurons can induce a significant release of glutamate in the LHb, leading to the occurrence of aversive behaviors [152]. Additionally, the activation of VTA glutamate-GABA neurons that contact LHb can result in both excitatory and inhibitory postsynaptic currents in the LHb, causing an inhibitory net effect that is thought to control the LHb glutamatergic input to the VTA [87]. In the case of VTA DA-GABA neurons projecting to the LHb, they appear to mainly release GABA to suppress the LHb output to the VTA and to promote reward-associated behaviors [145]. This effect is also supported by data from another study in which the authors showed that single-pulse stimulation of VTA DAergic neurons can result in a transient cessation of LHb neuronal activity, but VTA tetanic stimulation leads to an increase in LHb neuronal activity. This might reflect the differential signaling of both reward and aversive events through the VTA-to-LHb networks [49].

### 4.3. The Impact of the LHb–VTA Microcircuit Dynamics on Cognitive Activity

The direct manipulation of the LHb and its principal efferent source, the VTA, can clearly introduce strong changes in both the afferent and the efferent pathways involved in pain and cognitive information processing, further altering the balanced control leading to neuropsychiatric pathological disorders [3]. In Table 1, we summarize some of the most relevant studies highlighting the impact of LHb–VTA microcircuit manipulation on cognitive functions. As mentioned before, the LHb can encode negative reward value [9,31], which is believed to occur through the inhibition of VTA DA firing [19,20] and by a decrease in DA release in the NAc [151]. At the network level, both the LHb-to-VTA signaling and the reverse circuit, VTA-to-LHb, impact reward and aversion processing [87,145,152]. For example, the control exercised by the LHb–VTA pathway has been reported to be important for the performance of risk-based emotional tasks based on the precise phasic DA signals necessary to prepare future choice responses [37]. To evaluate the role and characterize the activity of the LHb and midbrain DA neurons, Matsumoto and colleagues (2007) [9] performed a study in primates using a saccade reward-related task. They found that whenever a reward was presented, there was silencing of LHb neurons, and during unrewarded trials, the LHb neurons fired phasically [9]. They also found that when a predicted reward was omitted, these neurons rapidly increased their firing activity in opposition to DA recorded neurons. Finally, they also reported that the short-term activation of the midbrain DA neurons induced by reward-predicting stimuli can occur before the transient inhibition of LHb neuronal activity, suggesting that DA neurons also convey positive reward signals to the LHb [9].

The ability to adapt and change responses during unpredictable events or to increase focus on a relevant stimulus while ignoring others is a key factor in the success of several emotional, executive, and cognitive functions [10,36,153,154,155]. These functions are strictly dependent on attentional levels during behavioral demands. LHb lesions have also been associated with the development of attentional deficits, leading to premature or impulsive responses during the performance of a five-choice serial reaction task [70]. These impairments are thought to be dependent on the malfunction of LHb control over DA centers. Using the same behavioral paradigm, a recent study also demonstrated the emergence of attentional deficits following the chemogenetic activation of VTA DA neurons [156]. As the LHb complex synaptic drive to local VTA neurons is mainly inhibitory, these studies support the hypothesis that increased VTA DA activity can be achieved indirectly through lesions of the LHb complex. This reinforces the idea that the LHb–VTA microcircuit plays a critical role in goal-directed actions during attention-demanding conditions. The LHb complex has also been associated with the modulation of behavioral avoidance responses. For example, Lammel and colleagues [38] optogenetically stimulated the LHb axonal terminals that terminate in the VTA, and they showed that this activation can lead to increased avoidance behavior [38]. Additionally, they found that these responses can be reverted if this activation is performed in combination with a local infusion of DA D1 receptor (D1r) antagonists in the mPFC, suggesting that the VTA input to the mPFC may also be important for avoidance responses. The stimulation of VTA has also been shown to increase active avoidance learning and the acquisition of novel strategies, whereas LHb stimulation has been shown to suppress the acquisition of novel strategies that are irrelevant to behavioral performance [80]. Interestingly, another study showed that LHb stimulation seems to have no significant impact on memory consolidation or retrieval but induces an impairment in the acquisition of avoidance learning [157]. In contrast, the LHb lesions appear to have a similar effect to that of VTA direct stimulation, as they improve avoidance learning [158]. Moreover, it has also been reported that continuous exposure to aversive stimuli increases LHb excitatory output onto the RMTg and that optogenetic activation of LHb glutamatergic terminals in the RMTg can promote active/passive and conditioned behavioral avoidance [159]. It is important to note that active avoidance behavior has rewarding components since it enables the subject to escape a noxious stimulus. Additionally, it might play a role in the cognitive processes required to learn the instrumental contingency. For example, learning the avoidance contingency also requires the animal to remember where the shock is given to actively avoid it, and it relies on contextual memory [160]. Consequently, a role in the DA system would be in place, with the activation of VTA, PFC, and NAc being reported when avoidance behavior was prompted [161]. In fact, there is an increased release of DA in the PFC during the acquisition phase of avoidance learning [162,163]. Overall, deficits or improvements in the acquisition phase of avoidance behavior might correlate with a role for DA in motivation and learning processes that are specifically related to working memory. This, in turn, can also be modulated by the LHb descending drive. Using a classical conditioning task in which freezing responses were evaluated, Chan and colleagues reported that impaired DA signaling in the LHb (through D1r activation or inhibition) affects the acquisition of contextual fear memory but not its consolidation or retrieval [164]. Additionally, LHb DA D1r pharmacological inactivation has also been associated with memory acquisition and retrieval deficits during the performance of a conditioned taste-aversion test, reflecting the importance of LHb DA signaling in aversion and memory [165]. Contextual memory is known to be hippocampal-dependent [166,167]. As there is no anatomical direct connectivity between the LHb and the hippocampal formation, it is assumed that this interplay between both structures is mediated mainly by indirect pathways, such as the VTA. In fact, it has been reported that the LHb might act as a controller, influencing the bidirectional interplay between the VTA and the hippocampus [140,168]. In this regard, LHb electrical stimulation has been shown to increase the release of DA in the hippocampal formation [169], developing an important role in the regulation of spatial working memory [170] and long-term memory [141]. This increase is strictly modulated by local DA D2r receptor activity via VTA projections [171]. The hippocampus receives sparse DAergic innervation from the VTA that regulates its local synaptic transmission, which is associated with mnemonic functions [172] and influences hippocampal-dependent behaviors [173]. Moreover, hippocampal DAergic system abnormalities in local circuits involved in working memory processing can explain pain-related performance deficits [119,174] and different pain sensitivity responses due to the action of D2r [175,176,177]. In this scope, a recent study from our laboratory has also observed that selective inhibition of LHb glutamatergic neurons projecting into the VTA enhances spatial working memory in inflammatory pain rats [178]. These studies suggest that LHb-to-VTA pathway dysfunction is an important factor for impairment of memory.

**Table 1:** Summary of studies investigating the role of LHb–VTA pathway on cognitive component.

Cognitive Variable	Experimental Model	Manipulation	Main Findings	Reference
Reward/ aversion	Primate	Electrical LHb Stimulation	LHb electrical stimulation elicits an inhibition of DA neurons; LHb input plays an important role in determining the reward-related activity of DA neurons	Matsumoto et al., 2007 [9]
Reward/ aversion	Rat	LHb electrical stimulation; Fasciculus retroflexus (Fr) lesion	LHb electrical stimulation elicits an inhibition of the VTA and substantia nigra (SN) DA neurons; Fr lesion attenuates LHb inhibition over DA neurons	Ji and Shepard, 2007 [19]
Reward/ aversion	Rat	Electrical/chemical modulation of the LHb	Inhibition of LHb increases DA release in the PFC, NAc, and dorsal striatum; LHb stimulation produces minimal opposite effects	Lecourtier et al., 2008 [151]
Reward/ aversion	VGLUT2-Cre mice	Activation of VTA glutamatergic neurons	VTA VGLUT2-mesohabenular neurons play a role in aversion by activating LHb glutamatergic neurons	Root et al., 2014 [152]
Reward/ aversion	TH-Cre mice	Activation of VTA TH-expressing neurons	This activation produces reward-related behavioral phenotypes that require GABA_A_ signaling in the LHb	Stamatakis and Stuber, 2012 [159]
Reward/ aversion	VGLUT2-Cre mice	Activation of VTA glutamatergic neurons	This activation induces positive reinforcement in instrumental behavioral assays by brief stimulation and avoidance in continuous stimulation	Yoo et al., 2016 [87]
Reward/aversion	Rat	Modulation of LHb, RMTg, or VTA activity	Dissection of the role of this brain area in the precise coordination of DA signals that regulate future reward–risk-based responses	Stopper et al., 2014 [37]
Attention	Rat	Bilateral LHb lesion	This lesion promotes attention deficits through premature or impulsive responses	Lecourtier and Kelly, 2005 [70]
Attention	TH-Cre rat	Chemogenetic activation of VTA or SN DA neurons	Activation of VTA/SN DA neurons promotes attention deficits, without affecting impulsivity	Boekhoudt et al., 2017 [156]
Avoidance	VGLUT2-Cre mice	Activation of LHb neurons projecting to VTA	This activation increases aversion after LHb light stimulation; aversion for light conditioned room blocked by D1r antagonist in mPFC	Lammel et al., 2012 [38]
Avoidance	Mice	VTA stimulation	VTA stimulation impairs avoidance acquisition, without affecting memory retrieval or motivation	Shumake et al., 2010 [80]
Avoidance	Gerbils	LHb stimulation	LHb stimulation impairs acquisition of avoidance learning, without affecting consolidation or retrieval	Ilango et al., 2013 [157]
Avoidance	Mice	Activation of LHb glutamatergic terminals in the RMTg	This activation promotes active/passive and conditioned behavioral avoidance	Stamatakis and Stuber, 2012 [159]
Contextual memory	Rat	Blockade or activation of LHb DA D1r	This manipulation impairs DA D1r signaling in the LHb and affects acquisition of contextual fear memory	Chan et al., 2017a [164]
Contextual memory	Rat	Blockade or activation of LHb DA D1r	This manipulation promotes anxiety-like behavior and decreases depressive-like behavior; impaired aversive memory acquisition	Chan et al., 2017a [164]
Contextual memory	Rat	Transient inactivation of VTA	This manipulation impairs hippocampal long-term memory	Ghanbarian and Motamedi, 2013 [141]

## 5. Future Directions and Concluding Remarks

The complex interactions between the LHb and VTA have attracted great interest with respect to their important role in the regulation of behavior, pathological chronic pain conditions, and impact on high-level cognitive functions. Here, we review the most recent advances in the understanding of the roles of this microcircuit, particularly those related to pain and cognition. From the survey of the current literature, it is clear that both brain areas are structurally and functionally connected and share dynamic bidirectional neural interactions. It is also well known that both structures participate in aversion and pain circuits, and that intrinsic dysfunctions affect pain processing and sensation. Thus, the LHb and VTA are important candidates and relevant targets to study in a broad range of pain-related pathologies associated with the sensorial, emotional and cognitive dimensions. New technologies such as optogenetics have the capability to achieve regional and cell-type neuronal activation, providing an unprecedented opportunity to probe the complexities of pain information processing and their impact on supraspinal circuits [179]. Somewhat surprisingly, only a few studies have taken advantage of these tools to understand the role of this pathway in pain-related dysfunctions. Considering the importance of this pathway, however, additional studies will move beyond these initial studies and use optogenetic tools to tackle unanswered questions regarding pain impact on circuitries not classically associated with pain processing.

## Data Availability

Not applicable.
